# Metacognition in Early Phase Psychosis: Toward Understanding Neural Substrates

**DOI:** 10.3390/ijms160714640

**Published:** 2015-06-29

**Authors:** Jenifer L. Vohs, Tom A. Hummer, Matthew G. Yung, Michael M. Francis, Paul H. Lysaker, Alan Breier

**Affiliations:** 1 Department of Psychiatry, Indiana University School of Medicine, Indianapolis, IN 46202, USA; E-Mails: thummer@iupui.edu (T.A.H.); mgyung@iupui.edu (M.G.Y.); mmfranci@iupui.edu (M.M.F.); plysaker@iupui.edu (P.H.L.); abreier@iupui.edu (A.B.); 2 Prevention and Recovery Center for Early Psychosis, Midtown Community Mental Health Centers, Eskenazi Hospital, Indianapolis, IN 46202, USA; 3 Department of Psychiatry, Roudebush VA Medical Center, Indianapolis, IN 46202, USA

**Keywords:** early psychosis, schizophrenia, metacognition, brain, magnetic resonance imaging

## Abstract

Individuals in the early phases of psychotic illness have disturbed metacognitive capacity, which has been linked to a number of poor outcomes. Little is known, however, about the neural systems associated with metacognition in this population. The purpose of this study was to elucidate the neuroanatomical correlates of metacognition. We anticipated that higher levels of metacognition may be dependent upon gray matter density (GMD) of regions within the prefrontal cortex. Examining whole-brain structure in 25 individuals with early phase psychosis, we found positive correlations between increased medial prefrontal cortex and ventral striatum GMD and higher metacognition. These findings represent an important step in understanding the path through which the biological correlates of psychotic illness may culminate into poor metacognition and, ultimately, disrupted functioning. Such a path will serve to validate and promote metacognition as a viable treatment target in early phase psychosis.

## 1. Introduction

Schizophrenia is a chronic and disabling psychiatric disorder [1], affecting greater than 20 million individuals worldwide [2]. It is associated with compromised daily functioning, as well as severe symptoms such as hallucinations, delusions, disordered thinking and odd behavior. Metacognitive dysfunction has gained increasing recognition for its importance to clinical course and outcome in schizophrenia and related psychotic illnesses. Indeed, impaired metacognition functions as an important and independent predictor of psychosocial function over time [3]. Although metacognition broadly refers to the process of thinking about cognitive states, it can be thought of as a spectrum of mental activities ranging from discrete (recognition of thoughts, feelings, and basic judgment) to more synthetic capacities. Because the neural correlates of synthetic metacognition have yet to be investigated, the present study focuses on this form of metacognition that, importantly, represents the ability to form and utilize integrated, complex mental representations of the self, others, and events in order to respond to life challenges [4,5].

Individuals with prolonged psychosis have compromised metacognitive capacity which has been linked to symptoms [5,6,7,8,9,10,11,12], learning difficulties [13], reduced motivation [14] and poor psychosocial functioning [10,15,16,17]. Importantly, these findings have recently been extended to include individuals in the early phase of psychosis (EPP), suggesting that metacognitive deficits emerge early in the disease process and may represent a risk factor for poorer outcomes [18,19,20,21]. Investigations focused on EPP capitalize upon the unique opportunity to study underlying illness pathophysiology with the goal of ultimately developing novel therapeutics and preventing the negative outcomes associated with chronic psychotic illness [22,23]. In particular, individuals with EPP typically have fewer comorbidities, shorter durations of antipsychotic treatment, and possibly lower severity of illness. Despite the advantages of studying EPP and the deleterious impact of impaired metacognition, our understanding of the neural substrates underlying metacognition in this population is limited. Such data is likely to be vital to identifying biological mechanisms of metacognition in EPP, informing the development of novel therapeutics that can ameliorate present and future dysfunction, and in providing an objective measure of cortical reorganization that can be used to monitor the progress and outcome of interventions [24].

Advances in technology over the last several decades have enabled increased ability to understand psychosis, moving across levels of analysis from basic science and the study of molecules to investigations of higher order disturbances in human consciousness. The purpose of this study was to elucidate the neural substrates underlying metacognitive capacity in EPP. One possible route to studying neural correlates of metacognition is to employ magnetic resonance imaging (MRI) techniques. Neuroimaging data allow for the examination of psychosis as a brain disorder with altered structure and function [25,26]. We performed a whole-brain grey matter density (GMD) analysis via voxel-based morphometry (VBM) to examine specific metacognition-morphometry associations. Volumetric measurements, such as VBM, are popular whole-brain analytical methods that can reveal regional brain abnormalities or associations with variables of interest, in an unbiased manner [27,28]. Meta-analyses, including studies of GMD in EPP and prolonged psychosis samples, have consistently demonstrated reduced volume in several regions. The most profoundly affected of these areas include the inferior and middle frontal cortices, anterior cingulate cortex, and insula [29,30,31,32]. Studies specifically examining discrete metacognitive processes suggest that areas of prefrontal cortex (PFC) may subserve a number of domains of metacognition [33,34]. Therefore, in the present investigation, we hypothesized that higher levels of metacognition would be dependent upon gray matter density (GMD), particularly in the prefrontal cortex.

## 2. Results and Discussion

The socio-demographic characteristics and average scores for measures of symptoms and metacognition are reported in Table 1. No associations with other demographic or symptom measures were revealed.

**Table 1:** Participant characteristics.

Characteristic	*M*	*SD*	*N* (%)
Male			20 (80%)
African American			18 (72%)
Age (years)	23.2	4.4	
Education			
Middle/junior high school			2 (8%)
High school, no degree			6 (24%)
High school, degree			7 (28%)
Some university courses			7 (28%)
Associate’s degree			1 (4%)
Bachelor’s degree			2 (8%)
SES *^a^*	2.7	1.1	
Age of onset of psychosis	21.0	4.8	
Duration of treatment (months) *^b^*	15.2	11.7	
Antipsychotic drug exposure *^c^*	164.1	187.5	
CGI-S	3.0	0.8	
PANSS Total	51.4	13.3	
Positive	12.0	4.9	
Negative	14.7	5.2	
Disorganized	15.1	5.1	
MAS-A Total	11.4	5.0	
Self Reflectivity	4.5	1.8	
Other Reflectivity	2.4	1.2	
Decentration	0.6	0.7	
Mastery	4.0	1.7	

*^a^* SES (socioeconomic status) measured using the Hollingshead Two Factor Index of Social Position (*Hollingshead* & Redlich, *1958*), which is comprised of an occupational and educational scale and ranges from 1 to 5 (higher index = higher SES); *^b^* calculated as number of months from date of first treatment for psychosis of date of imaging; *^c^* chlorpromazine (CPZ) equivalents in grams; CGI-S = Clinical Global Impression-Severity (range: 1 to 7, higher scores indicate worse global functioning); PANSS = Positive and Negative Syndrome Scale; MAS-A = Metacognitive Assessment Scale Abbreviated (a total score of metacognitive capacity); M = mean; SD = standard deviation.

### Metacognition and Whole Brain Gray Matter Density

Voxel based morphometry (VBM) analyses indicated that metacognition was positively correlated to gray matter density (GMD) in several locations (Table 2), with higher metacognition related to higher GMD. In the primary analysis, metacognition was associated with bilateral dorsal lateral (DLPFC) and medial (mPFC) prefrontal cortices, ventral striatum (VS), precentral gyrus, and anterior cingulate cortex (ACC). Correlations between the related brain areas and total metacognition and specific metacognitive domains are listed in Table 3.

**Figure 1:**
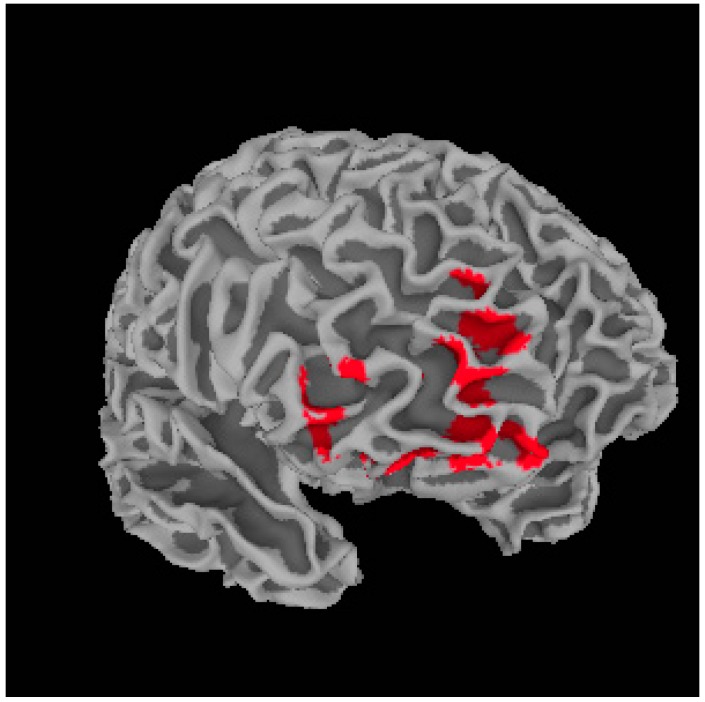
Illustration of significant positive relationship between the medial prefrontal cortex cluster and total metacognition score. Areas highlighted in red indicate regions of positive association.

**Figure 2:**
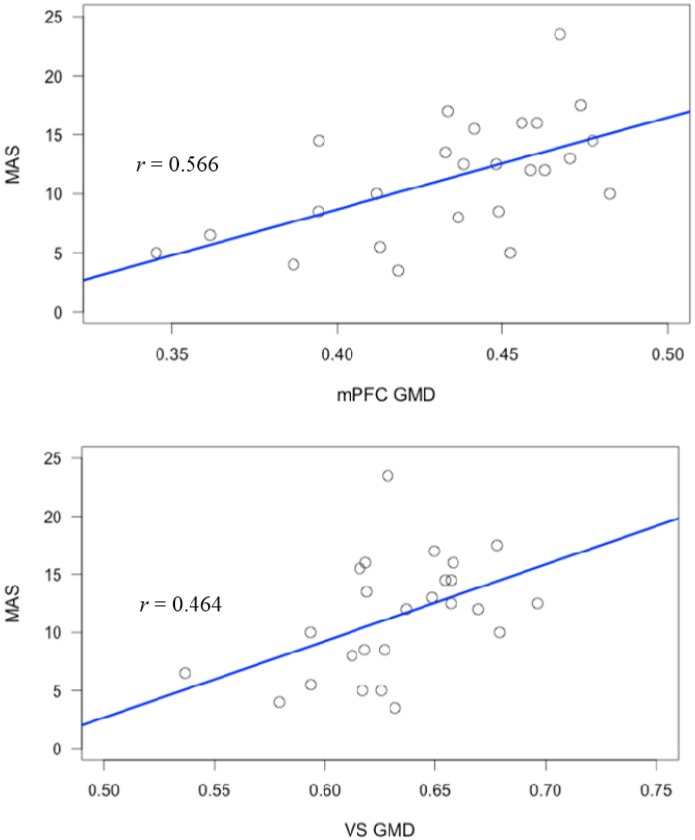
Scatterplots of GMD in significant clusters and group total metacognition score.

By including the Positive and Negative Syndrome Scale (PANSS) negative symptom cluster as a regressor, we demonstrated that the relationships of mPFC and VS with metacognition were not better accounted for by the association between total metacognition and negative symptoms (Table 4). Significant positive correlations were found to persist for the right mPFC (Table 5 and Figure 1) and VS (Table 5). These relationships were again positive suggesting increased GMD in these areas was associated with higher metacognition (Figure 2).

**Table 2:** Whole-brain gray matter volume analysis.

Region	R/L	BA	MNI Coordinates (*x*, *y*, *z*)	*t*-Value (Peak Voxel)	Cluster Size (Voxels)
Dorsal Lateral Prefrontal Cortex	L	10	−32, 47, 7	6.22	692
Dorsal Lateral Prefrontal Cortex	R	10/11	27, 34, 24	6.00	589
Medial Prefrontal Cortex	R	10/11	18, 62, 7	5.75	1684
Ventral Striatum	R	–	10, 16, −6	4.77	611
Precentral Gyrus	R	4	63, −1, 45	4.44	747
Anterior Cingulate Cortex	L	32	−3, 36, 27	4.39	860

Voxel-level *p* < 0.005 corrected at cluster-level to *p* < 0.05; BA = Brodmann area; MNI = Montreal Neurological Institute; R = Right; L = Left.

**Table 3:** Correlational analysis of gray matter density (GMD) in significant clusters and metacognition.

Region	MAS-A	Self-Reflect	Other-Reflect	Mastery
L.DLPFC	0.455 *	0.472 *	0.274	0.522 **
R.DLPFC	0.650 **	0.686 **	0.462 *	0.640 **
mPFC	0.565 **	0.606 **	0.411 *	0.572 **
VS	0.506 **	0.405 *	0.420 *	0.524 **
Precentral Gyrus	0.260	0.275	0.087	0.312
ACC	0.568 **	0.599 **	0.455 *	0.563 **

All values represent Pearson correlation coefficients (*r*). GMD = gray matter density; MAS-A = Metacognitive Assessment Scale Abbreviated (a total score of metacognitive capacity); L.DLFPF = dorsal lateral prefrontal cortex; R.DLPFC = dorsal lateral prefrontal cortex; mPFC = medial lateral prefrontal cortex; VS = ventral striatum; ACC = anterior cinculate cortex; * *p* < 0.05; ** *p* < 0.01.

**Table 4:** Whole-brain gray matter volume results, covarying for PANSS negative scores.

Region	R/L	BA	MNI Coordinates (*x*, *y*, *z*)	*t*-Value (Peak Voxel)	Cluster Size (Voxels)
Medial prefrontal cortex	R	9/10	18, 63, 7	4.07	2482

Voxel-level *p* < 0.005 corrected at cluster-level to *p* < 0.05; BA = Brodmann area; MNI = montreal neurological institute; R = right; L = left; PANSS = Positive and Negative Syndrome Scale.

**Table 5:** Correlational analysis of GMD in significant clusters (covarying for PANSS negative scores) and metacognition.

Region	MAS-A	Self-Reflect	Other-Reflect	Mastery
mPFC	0.566 **	0.605 **	0.424 *	0.564 **
VS	0.464 *	0.451 *	0.367	0.513 **

All values represent Pearson correlation coefficients (*r*). GMD = gray matter density; MAS-A = Metacognitive Assessment Scale Abbreviated (a total score of metacognitive capacity); * *p* < 0.05, ** *p* < 0.01.

Taken together, the above findings support our hypothesis that metacognition as measured by the Metacognitive Assessment Scale Abbreviated (MAS-A) is subserved by specific neuroanatomical structures. Specifically, even after controlling for negative symptoms [5], higher metacognition scores were linked to greater GMD in both right mPFC and ventral striatum. Though the methods and data presented here preclude causal inference, the findings do suggest that these areas likely represent neuroanatomical sites required for metacognitive processing in EPP.

The implication of mPFC in synthetic metacognition is consistent with a number of studies that suggest mPFC may play an important role in metacognitive processes [33]. These results are also consistent with widely accepted research that has shown that the mPFC plays an important part in self referential [35] and socio-emotional processing [36]; functions necessary for forming and integrating complex ideas about the self and others. Self referential processing involves linking information to be remembered with knowledge of the self [35,37]. Recent evidence also suggests however that the functions of the mPFC may be more clearly defined by domain and the area within mPFC [34]. Moran and colleagues [38] showed that the dorsal mPFC was engaged when participants thought about characteristics relevant to both self and others, while ventral mPFC was specifically engaged when individuals were cued with information specific to themselves. In the present study, metacognition was not related to a specific area of mPFC. One potential explanation for our more inclusive finding is that synthetic metacognition requires integration of knowledge of the self and others in order to form meaningful ideas about personal and social experiences.

The second finding, that total metacognition was associated with GMD of the ventral striatum (VS), was not an a priori prediction but nonetheless may have important implications. The VS is thought to consist primarily of the nucleus accumbens and olfactoray tubercle [39], with the nucleus accumbens often considered the key structure [40]. The striatum is densely innervated by dopaminergic connections [41,42] with distinct functionality. Data drawn from the Parkinson’s disease literature, for instance, suggests that dopamine dysregulation correlates with aberrant patterns of neural activity in cortical networks involving VS [43]. The VS specifically receives afferent input from the ventral tegmental area with reciprocal connections with cortical (anterior cingulate, orbitofrontal, and anterior temporal) and limbic (hippocampus, insula, amygdala, hypothalamus) regions [43]. Such connections are thought to be involved in a number of cognitive processes that may be vital to metacognition [5], including associating events across time, orientation, implicit and explicit learning, temporal binding of experience, and assessment of the motivational state of an individual [43]. Imaging studies suggest that VS is also differentially active during novelty or salience tasks [44], as well as those involving social rewards [45] and encoding facial emotion [46,47]. These processes could be expected to support the complex integration of personal and social information as needed for metacognition. Moreover, if these functions of the VS were compromised due to reduced GMD, it could provide a neurobiological basis for the supposition that loss of metacognitive capacity leaves persons without a larger sense of the meaning of past and current events and a compromised ability to manage life challenges. Further research, using functional measures, will be needed however to elucidate the potential role of the striatum and connected structures in metacognitive processing.

As mentioned above, individuals in the early phase of psychosis appear to experience metacognitive deficits [18,19] that may even exceed what is observed in the later phases of illness [20], but there is a paucity of data about possible neural substrates. An important implication of the present work is that, by furthering understanding of the neurobiological substrates of metacognition in EPP, a novel early intervention target could be validated and promoted [48,49]. For instance, a number of neuroimaging studies have shown that psychological interventions for psychosis, such as cognitive remediation and cognitive behavior therapy, result in functional normalization of fronto-cortical areas [24]. Continued study of such relationships seems particularly vital in EPP since early intervention may reduce chronic dysfunction and improve outcomes [22,23].

**Figure 3:**
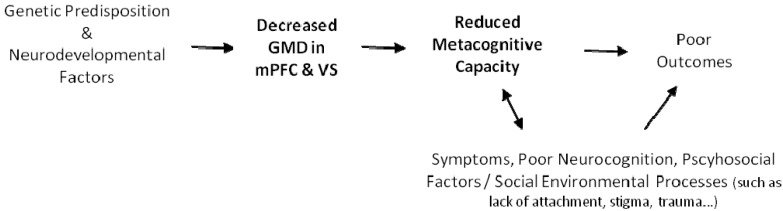
A path from gene to brain to dysfunctional behavior in schizophrenia.

Early scholars described psychosis as an interruption in goal directed behavior secondary to a complex, biologically based decomposition and unbinding of associations that collapsed higher order understanding of self and others [50]. However, over several decades a more detailed understanding of the sequence of events which converge in psychotic illness to result in dysfunction has emerged. Genetic vulnerabilities for psychosis have, for instance, been linked with abnormalities in brain development, which are reflected in specific cognitive deficits [51,52,53]. Albeit simplified, we propose a model that draws from this work and also offers a possible path from aberrant brain structure (reduced GMD in mPFC and VS) to the dysfunction associated with EPP (see Figure 3). Such a model, if supported with further research, would represent another step in moving toward understanding the neuroantomical underpinnings of higher order metacognitive process that are directly linked to functioning and outcomes for individuals with psychotic illness [3].

A number of caveats should be considered when interpreting the findings of this study. First, the relatively modest sample used here included primarily young, African American males within 5 years of initial diagnosis of psychotic illness. It is therefore possible that a sample with more females or individuals of varied ethnic backgrounds, or perhaps even those experiencing their very first acute episode of illness, may have different results. Because there was no comparison group in this study, it is also difficult to say exactly how the observed metacognitive deficits are related to structural abnormalities. It is possible that the observed link between the study variables was due to some other unforeseen population-specific factor. The current study did account for factors such as antipsychotic drug exposure or current symptomatology and other potential confounds related to volumetric measurements such as VBM (registration differences, size of the smoothing kernel, shape differences that arise from systematic registration errors during spatial normalization, and image noise) are difficult to control. In terms of VBM, blurring is 3 dimensional and therefore does not respect boundaries along tissue classes, leading to increased probability of either diluting existing signal or misinterpreting boundary shift as signal. Additionally, only a single imaging analysis technique was employed in the present study. Other techniques, such as Diffusion Tensor Imaging (DTI) analysis may yield insight into to how white matter integrity affects metacognitive capacity.

In addition to these limitations, a number of questions remain. Medial PFC and ventral striatum GMD is linked to metacognitive capacity, but exactly how does this occur? Do such neurobiological alterations mediate the impact of metacognitive deficits on other aspects of the illness and, ultimately, behavior? Do the structures that subserve metacognition also play a role in other important psychological phenomenon, such as poor awareness of illness that are thought to be linked to metacognitive processing? Answers to these and other questions seem essential in order to develop fully elucidate neural correlates and treatments that adequately intervene at the level most proximate to function.

## 3. Experimental Section

### 3.1. Study Participants

Twenty-five individuals with EPP were recruited through the Indiana University Psychotic Disorders Program, within the Indiana University School of Medicine. All study procedures were completed via two concurrent studies both approved by the Indiana University Institutional Review Board, protocol numbers 1112007648 (initially approved 9 February 2012) and 1011002975 (initially approved 14 September 2010). After receiving an explanation of study procedures, subjects gave their written informed consent prior to enrollment. At a baseline visit, demographic information and medical history were recorded. Patients were ages 18–35, within the first five years of psychotic illness onset, including diagnoses of schizophrenia, schizophreniform disorder, schizoaffective disorder, or Psychotic disorder not otherwise specified, as confirmed by a trained psychiatrist or psychologist via clinical interview and/or administration of the Structured Clinical Interview for Diagnosis (SCID) for the Diagnostic and Statistical Manual of Mental Disorders-IV (DSM-IV) [54]. All patients were on antipsychotic medication at the time of testing (mean chlorpromazine equivalent = 164.1, standard deviation = 187.5). Exclusionary criteria included active substance abuse or dependence within three months of testing, a history of total IQ less than 70, as documented in the medical record, or MR incompatibility. Further details are listed in Table 1.

### 3.2. Metacognition and Symptom Assessment

#### 3.2.1. Measurement of Metacognition

Metacognitive capacity was assessed in a two-step process. First a narrative about self and illness was elicited using the Indiana Psychiatric Illness Interview (IPII) [55]. The IPII is a semi-structured interview which generally takes between 30 and 60 min to complete. Responses are audio recorded, transcribed, and then rated by trained investigators using the Metacognition Assessment Scale Abbreviated (MAS-A) [10]. The MAS-A was cooperatively developed with the authors of the MAS [56] in order to develop an instrument to assess metacognition as manifest in personal narratives. Good inter-rater reliability (intraclass correlation = 0.82) and validity has been presented elsewhere [3]. The MAS-A includes subscales in several domains: “Understanding of one’s own mind” or the comprehension of one’s own mental states (range 0—no awareness of own mental states, to 9—recognizes thoughts and emotions across larger life story); “Understanding of others’ minds” (range 0—no awareness of others mental states to 7—complete description of others, across larger life story); “Decentration” or the ability to see the world as existing with others having independent motives (range 0—views the self as the center of all events, to 3—understands others have separate, independent lives from individuals and that things unfold as a result of larger, complex factors); and “Mastery” or the ability to work through one’s representations and mental states to implement effective action strategies in order to accomplish cognitive tasks or cope with problematic mental states (range 0—unaware of own psychological problem, to 9—able to respond to psychological problems based on knowledge of larger life understanding of self and others). For the purposes of this study, the domains were not only examined independently but also combined to form a total score for metacognitive capacity (range 0 to 28). Higher scores indicate higher metacognition.

#### 3.2.2. Measurement of Symptoms

Symptoms were assessed via the Positive and Negative Syndrome Scale (PANSS), which is a 30-item scale designed to be completed by a trained rater at the conclusion of an interview and chart review [57]. Previous work has shown that MAS-A ratings, and thus metacognitive capacity, are correlated with levels of negative symptomatology [9]. Therefore, the Negative subscale, of the PANSS factor analytically derived components [58], was utilized in statistical analyses. All PANSS assessments were completed by well-trained, supervised masters-level clinicians with adequate inter-rater reliability (intraclass correlations: 0.85 to 0.93).

### 3.3. Neuroimaging Acquisition and Analysis

#### Structural Magnetic Resonance Imaging (MRI) Data Acquisition and Analysis

At the imaging visit, patients underwent a magnetic resonance imaging (MRI) scan on a 3T Tim Trio scanner (Siemens, Erlangen, Germany) with a 32-channel phased array head coil. Brain structure was characterized with a high-resolution T1-weighted whole-brain magnetization prepared rapid gradient echo (MPRAGE) scan with the following parameters: 160 3D sagittal slices, echo time/repeat time/inversion time = 2.91/2300/900 ms; slice resolution = 100%; Echo spacing = 7.7 ms, flip angle = 9°, field of view = 240 × 256 mm, voxel size = 1 × 1 × 1.2 mm.

Gray matter density was quantified using unmodulated, normalized data using VBM, the VBM8 toolbox [59] within the Statistical Parametric Mapping software (SPM8) [60] package, which was run via Matlab 2012a (MathWorks Inc., Natick, MA, USA). A standard protocol within VBM8 was followed for assessing the relationship of metacognition scores with structural characteristics. In this process, MRI images were first spatially normalized to the ICBM-152 standard template [61] and resampled to isotropic 1.5 × 1.5 × 1.5 mm voxels. An automated procedure then segmented each image into gray matter, white matter, and cerebrospinal fluid [28]. Normalized data were then smoothed with an 8-mm full-width-at-half-maximum (FWHM) isotropic Gaussian kernel.

To quantify the relationship of gray matter density to metacognitive capacity, we conducted a multiple regression analysis, with age, sex (as a dummy variable), and total intracranial volume as regressors along with MAS-A total scores. Given that metacognition is associated with negative symptoms in schizophrenia [5], we conducted a separate regression, with identical regressors but also including PANSS negative symptom scores. A statistical threshold was set at a voxel-level *p* < 0.005, with a cluster-level family-wise error (FWE) correction of *p* < 0.05 to correct for multiple comparisons. Data from significant clusters were extracted using the MarsBaR (toolbox (http://marsbar.sourceforge.net) within SPM8) for visualization and *post-hoc* analyses with the Statistical Package for the Social Sciences (SPSS) 22 (IBM Corp., Armonk, NY, USA).

## 4. Conclusions

This is the first study to examine the neural correlates of metacognition, as measured by the MAS-A, in EPP. The present study demonstrated that increased GMD of the mPFC and VS is associated with higher levels of metacognition. The correlations among these areas and metacognitive processing fit the existing literature on the functions currently believed to be mediated by both areas. Such associations represent an important step in understanding the neurobiological underpinnings of disrupted metacognition in EPP, which ultimately culminates into poor outcomes. It also promotes and validates metacognition as an important treatment target in early intervention for psychosis and provides neurobiological marker(s) to track changes due to psychotherapeutic interventions (e.g., metacognitive therapy or remediation) and may lead to more accurate predictions of real-life/clinical outcomes.
